# Early Transcriptomic Changes upon Thalidomide Exposure Influence the Later Neuronal Development in Human Embryonic Stem Cell-Derived Spheres

**DOI:** 10.3390/ijms21155564

**Published:** 2020-08-03

**Authors:** Mami Kikegawa, Xian-Yang Qin, Tomohiro Ito, Hiromi Nishikawa, Hiroko Nansai, Hideko Sone

**Affiliations:** 1 Laboratory of Kampo Pharmacology, Yokohama University of Pharmacy, Yokohama 245-0066, Japan; m.takigawa@hamayaku.ac.jp; 2 Liver Cancer Prevention Research Unit, RIKEN Cluster for Pioneering Research, Wako, Saitama 351-0198, Japan; xyqin@riken.jp; 3 Center for Health and Environmental Risk Research, National Institute for Environmental Studies, Tsukuba, Ibaraki 305-8506, Japan; itotomo@nies.go.jp (T.I.); zahahi@yahoo.co.jp (H.N.); 4 Department of Psychiatry and Behavioral Science, Kanazawa University School of Medicine, Kanazawa, Ishikawa 920-0942, Japan; hiromi.a.rac@gmail.com; 5 Environmental Health and Prevention Research Unit, Yokohama University of Pharmacy, Yokohama 245-0066, Japan

**Keywords:** thalidomide, environmental chemicals, neuronal differentiations, human embryonic stem cells, computational network analysis

## Abstract

Stress in early life has been linked with the development of late-life neurological disorders. Early developmental age is potentially sensitive to several environmental chemicals such as alcohol, drugs, food contaminants, or air pollutants. The recent advances using three-dimensional neural sphere cultures derived from pluripotent stem cells have provided insights into the etiology of neurological diseases and new therapeutic strategies for assessing chemical safety. In this study, we investigated the neurodevelopmental effects of exposure to thalidomide (TMD); 2,2′,4,4′-tetrabromodiphenyl ether; bisphenol A; and 4-hydroxy-2,2′,3,4′,5,5′,6-heptachlorobiphenyl using a human embryonic stem cell (hESC)-derived sphere model. We exposed each chemical to the spheres and conducted a combinational analysis of global gene expression profiling using microarray at the early stage and morphological examination of neural differentiation at the later stage to understand the molecular events underlying the development of hESC-derived spheres. Among the four chemicals, TMD exposure especially influenced the differentiation of spheres into neuronal cells. Transcriptomic analysis and functional annotation identified specific genes that are TMD-induced and associated with ERK and synaptic signaling pathways. Computational network analysis predicted that TMD induced the expression of DNA-binding protein inhibitor ID2, which plays an important role in neuronal development. These findings provide direct evidence that early transcriptomic changes during differentiation of hESCs upon exposure to TMD influence neuronal development in the later stages.

## 1. Introduction

There has been worldwide concern over the increasing number of patients with depression and children with developmental disorders [1,2]. Recent studies suggest that the increasing prevalence of developmental disability in children is due to not only genetic factors but also some environmental factors [3]. Environmental factors including exposure to chemicals such as pesticides and air pollutants during the developmental age could play a role in the development of neurodevelopmental diseases [4,5]. In addition, the developing brain has been shown to be more sensitive to environmentally hazardous chemicals than the adult brain [6,7]. Recent studies indicate that in vitro models are starting to replace traditional in vivo models for evaluation of the effects of external substances on fetuses and for the assessment of neurotoxicity to chemical exposure; this alternative approach can bridge the mechanistic gap between humans and animals and can be used to elucidate new therapeutic approaches [8,9,10]. Therefore, systems using human embryonic stem cells (hESCs) and human induced pluripotent stem cells (hiPSCs) have been developed to directly predict human risks; the development of these systems would provide important information to elucidate the neurodevelopmental toxicities of numerous environmental chemicals.

We previously developed in vitro models using hESCs for studying the neurodevelopmental toxicities caused by various environmental pollutants [11,12,13]. Our previous work also showed that thalidomide (TMD) inhibits the development of dopaminergic neurons from neuronal progenitor cells [14]. Anti-depressant-like effects of maternal exposure to TMD was also observed in mice [15,16]. Early exposure to TMD in the pregnant period caused developmental abnormality in the human brain [17,18]. TMD is currently used to treat multiple myeloma, while it is a known teratogen and neurodevelopmental toxicant. Although it was recently shown that the degradation of spalt-like transcription factor 4 (SALL4) may be an essential component of TMD-induced teratogenicity that causes severe birth defects in the fetus [19,20], this mechanism is not enough to explain developmental neurotoxicity of TMD observed in in vitro and in vivo experiments. In addition, 2,2′,4,4′-tetrabromodiphenyl ether (BDE-47), bisphenol A (BPA), and 4-hydroxy-2,2′,3,4′,5,5′,6-heptachlorobiphenyl (4OH-PCB187) were also included in this study in comparison to TMD, since they show a high association with neuronal developmental disorders in epidemiologic studies and in animal and cellular experiments [21,22,23,24]. Grandjean and Landrigan suggested polybrominated diphenyl ethers (PBDEs) as one group of newly recognized developmental neurotoxicants including organophosphate pesticides, herbicides, fungicides and manganese. Bisphenol A is also suggested as another suspected developmental neurotoxicant [25]. 4OH-PCB187 is one of main metabolites for PCBs and they concentration was found in blood at a higher concentration, rather than other congeners [26]. PCB and their metabolites are very similar to the structures of PBDEs and their hydroxyl metabolites [27].

Collectively, to understand the molecular events underlying the neurodevelopmental effects of environmental chemicals including drugs, endocrine disruptors, and flame retardants, we have studied the effects of TMD and three environmental pollutants including BDE-47, BPA, and 4OH-PCB187 on global gene expression during neurosphere formation and during the following differentiation into neuronal cells.

## 2. Results and Discussion

### 2.1. Morphological Analysis of the Effect of Chemical Exposure at the Early Stage of Development on the Neuronal Differentiation from hESC-Derived Spheres

**Figure 1:**
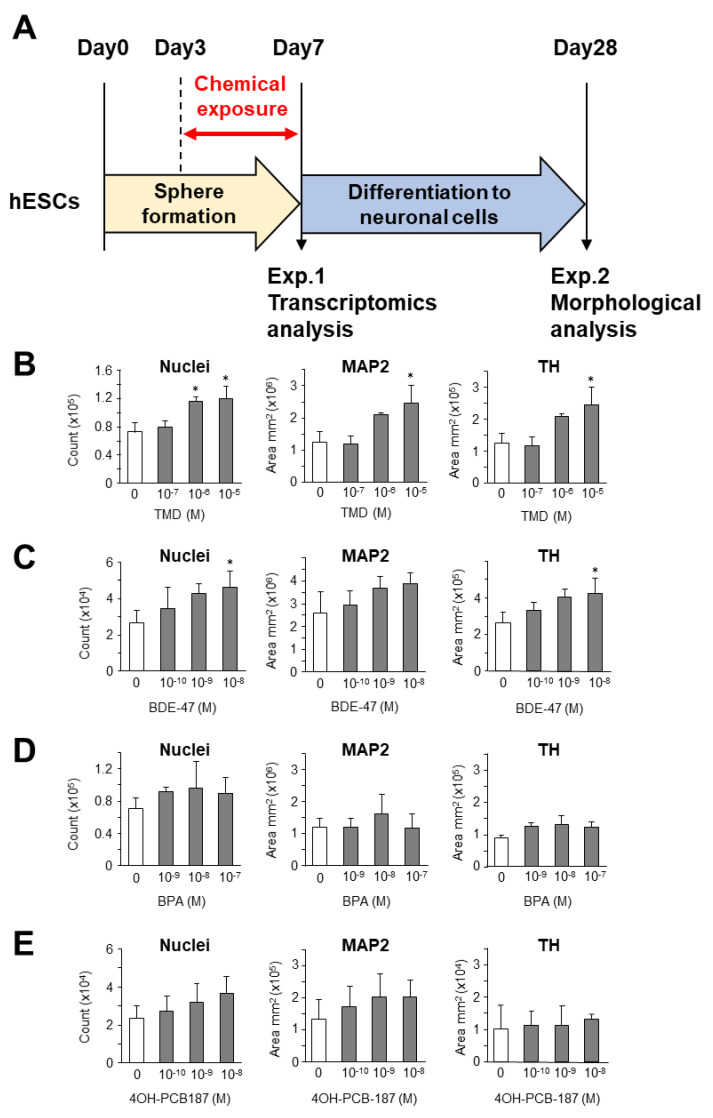
Morphological analysis of the effect of chemical exposure on neuronal differentiation from human embryonic stem cell (hESC)-derived spheres. (**A**) Experimental protocol of sphere formation and neuronal differentiation from hESCs. Dose-dependent effects of exposure to (**B**) thalidomide (TMD), (**C**) 2,2′,4,4′-tetrabromodiphenyl ether (BDE-47), (**D**) bisphenol A (BPA), and (**E**) 4-hydroxy-2,2′,3,4′,5,5′,6-heptachlorobiphenyl (4OH-PCB187) at the early stage of development on the number of cell nuclei and microtubule-associated protein 2 (MAP2)-positive and tyrosine hydroxylase (TH)-positive areas during neuronal differentiation from hESC-derived spheres. The data are represented as mean ± standard deviation of at least three biological replicates. * *p* < 0.05, analysis of variance (ANOVA) test.

In order to investigate the neurodevelopmental effects, we generated a protocol for sphere formation from hESCs and differentiation to neuronal cells (Figure 1A). In this model, we confirmed the differentiation of spheres into neuronal cells on Day 28 by immunostaining with anti-microtubule-associated protein 2 (MAP2) and anti-tyrosine hydroxylase (TH) antibodies (Figure S1A). Next, we exposed the spheres to each chemical, and examined the effects of chemical exposure on differentiation potency. Our results showed that TMD significantly increased the total cell numbers, and the presence of MAP2-positive and TH-positive neuronal cells in a dose-dependent manner (Figure 1B and Figure S1A). Slight induction of TH-positive cells but not MAP2-positive cells was observed with exposure to 10^−8^ M BDE-47 (Figure 1C). No adverse effects were observed upon exposure to BPA and 4OH-PCB187 (Figure 1D,E, respectively). Furthermore, the normalized MAP2-positive area and TH-positive area with the total cell number showed that the neuronal differentiation-promoting effect was only observed with TMD (Figure S1B). However, there was no significant change in TH/MAP2 ratio, which may be due to the promotion of nervous system differentiation or the expansion of the sphere itself. At that expansion stage, the TMD-treated cells in spheres may not be involved in promoting or suppressing the differentiation of TH.

The increase in number of neuronal cells upon TMD exposure was not consistent with another study that has investigated high-concentration exposure of this chemical [28]. These results indicate that the early exposure to TMD in vitro promotes neuronal development at a later stage of hESCs, contrary to previous reports that hESC-derived neuronal progenitor cells were exposed at the later stage [14]. The increase in TH-neuronal cells in the BDE-47 exposure was consistent with another study that the similar dose range of BDE-47 slightly increased the TUBB3 expression associated with dopaminergic neuron [29]. These results highlighted the importance to evaluate risk of environmental chemicals, such as the effect on neuronal development, in the hESC-derived sphere model at realistic blood concentration levels and timing of exposure.

### 2.2. Transcriptional Analysis of the Effect of Chemical Exposure at the Early Stage of Neuronal Development Derived from hESCs

**Figure 2:**
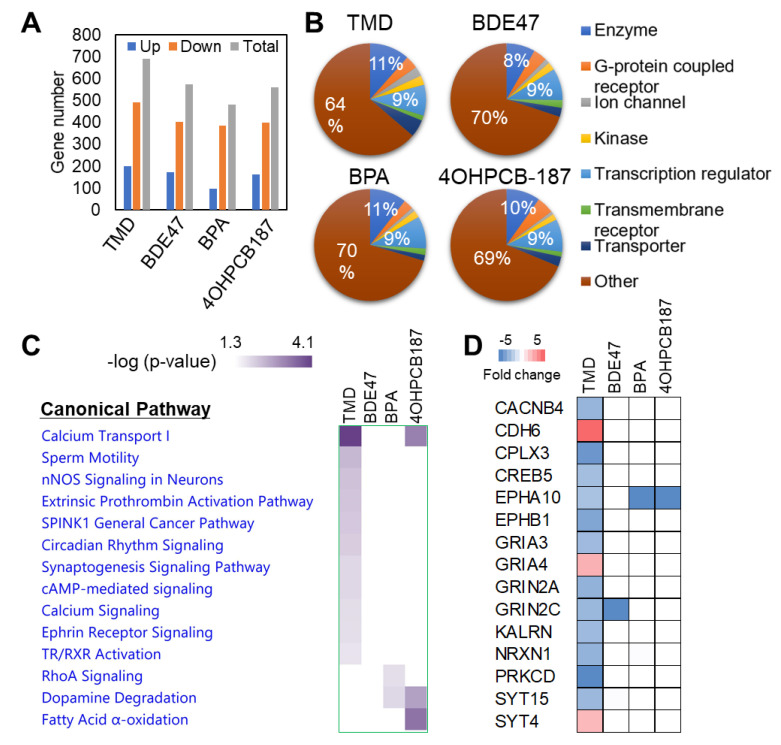
Transcriptome analysis of the effect of chemical exposure on gene expression in hESC-derived spheres. (**A**) Summary of the number and (**B**) functional annotation of differentially expressed genes in hESC-derived spheres after exposure to 1 μM TMD, 0.01 μM BDE47, 0.1 μM BPA, and 0.01 μM 4OH-PCB187. (**C**) Canonical pathway analysis of differentially expressed genes generated using the knowledge-based functional analysis software Ingenuity Pathways Analysis (IPA). (**D**) Expression of enriched genes involved in the synaptogenesis signaling pathway.

Next, to explore the molecular mechanism underlying the effects of chemical exposure on the differentiation of hESCs, we performed microarray-based transcriptome analysis to examine the global gene expression changes at the sphere stage (on Day 7). The chemical concentration for the microarray analysis was determined according to the results of the morphological analysis. For BPA and PCB, the highest concentrations were chosen since there were no significant effects at any concentration. For TMD and BDE, the lowest observed effect concentrations were chosen. Each chemical induced the changes of gene expression at a comparable level. For this comparison, only genes with a fold change of more than two compared to vehicle control were considered (Figure 2A). Further, exposure to different chemicals showed that there are similar functional annotations of differentially expressed genes (Figure 2B). The percentage of differentially expressed transcriptional factors was approximately 9% in all chemical cases (Figure 2B). These results suggest that our experimental strategy was able to capture the similar impact of different chemical exposures on transcriptomic changes.

To further understand the functional difference upon each different chemical exposure, the differentially expressed genes were imported into the IPA program. Canonical pathway analysis showed the strongest impact of TMD exposure in regulating multiple biological pathways as compared to other chemicals (Figure 2C). In accordance with the findings in the morphological analysis (Figure 1B), the gene expression of *MAP2* was selectively induced upon TMD exposure (Figure S1C). In addition, among the differentially expressed genes induced upon TMD exposure, we could identify an enrichment of genes encoding molecules regulating neuronal functions, such as “nNOS Signaling in Neurons”, “Extrinsic Prothrombin Activation Pathway”, “Circadian Rhythm Signaling”, and “Synaptogenesis Signaling Pathway” (Figure 2C). Gene expression profiling showed that TMD exposure selectively modulated the expression of *CACNB4, CDH6, CPLX3, CREB5, EPHB1, GRIA3, GRIA4, GRIN2A, KALRN, NRXN1, PRKCD, SYT15,* and *SYT4* (Figure 2D and Figure S2).

### 2.3. TMD-Specific Effect on Gene Expression at the Early Stage of Neuronal Development Derived from hESCs

**Figure 3:**
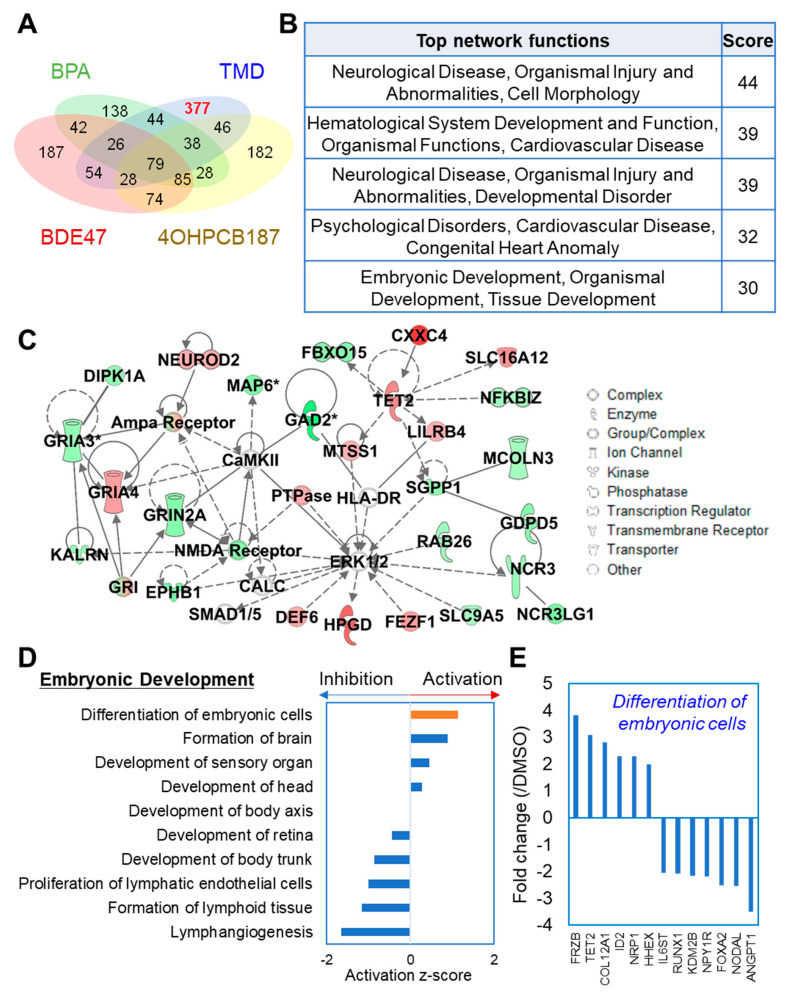
TMD-specific effect on gene expression in hESC-derived spheres. (**A**) Comparison of differentially expressed genes in hESC-derived spheres upon exposure to 1 μM TMD, 0.01 μM BDE47, 0.1 μM BPA, or 0.01 μM 4OH-PCB187. The red number indicates TMD-specific genes. (**B**) The biological functions of the top populated networks generated in IPA associated with differentially expressed genes specifically induced by TMD exposure. (**C**) A representative network related to neurogenesis entitled “Neurological Disease, Organismal Injury and Abnormalities, Cell Morphology”. Upregulated genes under control of TMD are indicated in red symbols, downregulated genes under control of TMD indicated in green symbols, and genes that were not annotated in this study, but are part of this network were indicated in white symbols. Solid lines indicate direct relationships, and dotted lines indicate indirect gene-gene relationships within the represented network. (**D**) The biological pathways of embryonic development generated in IPA were ranked by z-score, which can be used to find the likely regulating molecules based on a statistically significant pattern match of up- and down-regulation, and also to predict the activation state (activated or inhibited) of a putative regulator. (**E**) Expression of enriched genes involved in the “Differentiation of embryonic cells” pathway.

The differentially expressed genes that are induced by TMD but not the other chemicals were selected if they have a fold change higher than the fold change of 2. TMD-specific genes with 377 candidates were selected after the comparison between TMD and each of the three chemicals correspondingly (Figure 3A and Table S1) and was imported into IPA for pathway analysis. Interestingly, top network function analysis showed that a wide range of signaling pathways associated with neurological disease and embryonic development were specifically affected by TMD exposure (Figure 3B). The most highly populated network entitled “Neurological Disease, Organismal Injury and Abnormalities, Cell Morphology” indicated the central role of extracellular signal-regulated kinases ERK1/2 in controlling the transcriptional response of hESC-derived sphere in response to TMD exposure (Figure 3C). Further upstream causal network analysis confirmed that TMD might be exerting its neurodevelopmental effect via the suppression of ERK1/2 activation (Figure S3). In accordance with our findings in the hESC-derived sphere model, a previous study in the human neural stem cell model showed that inhibition of ERK by chemical inhibitors promoted neuronal generation, especially of TH-positive neurons [30]. To further understand the neurodevelopmental effect of TMD, the effect of TMD on biological pathways related to embryonic development was evaluated according to the activation z-score, which is a statistical measure in IPA and can be used to predict the activation state (activated or inhibited) of a biological molecule or function based on a statistically significant pattern match of up- and down-regulated gene expression [31]. Interestingly, the function of “Differentiation of embryonic cells” was predicted to be activated by TMD based on the expression of enriched genes such as FRZB, TET2, CoL12A1, ID2, NRP1, HHEX, IL6ST, RUNX1, KDM2B, NPY1R, FOXA2, NODAL, and ANGPT1 (Figure 3D,E).

### 2.4. Integrated Network Analysis of Transcriptional and Morphological Changes during Neuronal Differentiation from hESC-Derived Spheres

**Figure 4:**
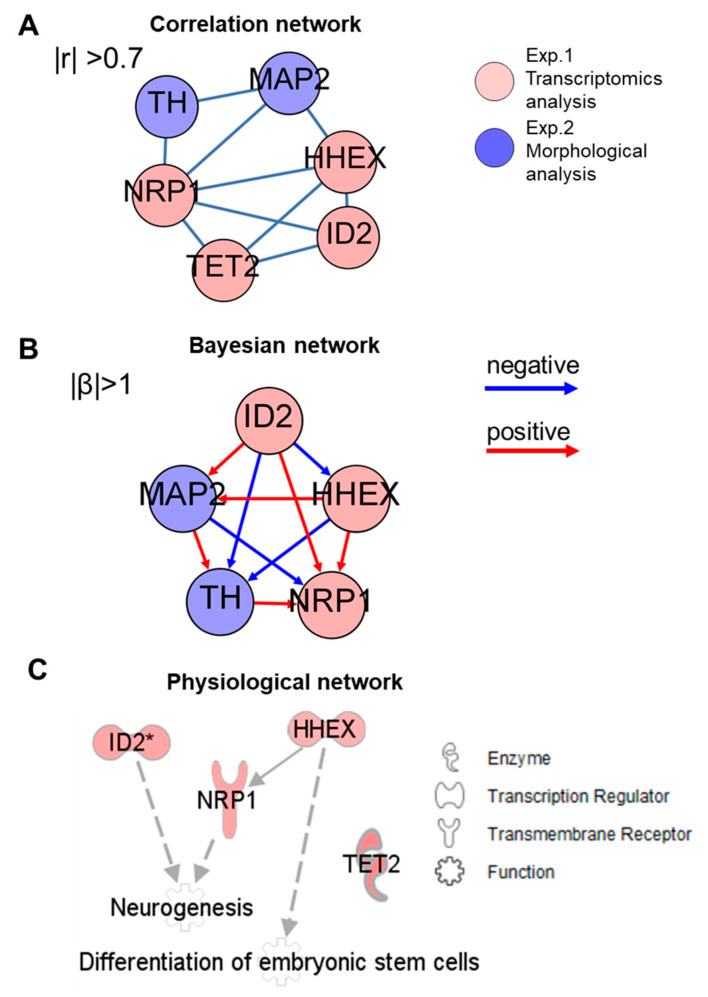
Integrated network analysis of transcriptomic and morphological changes during neuronal differentiation from hESC-derived spheres. (**A**) Pearson correlation analysis. The links with an absolute Pearson correlation coefficient of more than 0.7 are shown. The network was visualized using Gephi software. (**B**) Bayesian network analysis. The network was generated based on the TAO-Gen algorithm using the web-based RX-Taogen software and was visualized using Gephi software. The β-value of the Bayesian model is expressed as a red arrow if positively relative (β > 1), and a blue arrow if negatively relative (β < 1). (**C**) Physiological network generated using the Ingenuity Knowledge Base of the IPA software. Upregulated genes induced by TMD exposure are indicated in red symbols that are representing molecules. The solid line indicates direct interactions gene-gene association. Dot lines indicate indirect interactions to “Neurogenesis” or “Differentiation of embryonic stem cells”.

Finally, integrated network analysis was performed to determine the connection between the genetic and morphological actions of TMD during neuronal differentiation from hESC-derived spheres. Candidate feature genes involved in the function of “Differentiation of embryonic cells” (Figure 3E) with correctly predicted expression patterns in comparison with previous findings in the Ingenuity Knowledge Base were selected for network analysis (Table S2). As the result, expression data of four genes (TET2, HHEX, ID2 and NRP1) and two morphological measures of TH and MAP2 staining after chemical exposure were used for network analysis using three approaches: correlation-based network analysis, Bayesian network analysis and physiological network analysis. The correlation network was generated by calculating the Pearson correlation coefficient between each pair of gene and morphological parameters (Figure 4A). Either the four genes or the two morphological measures were correlated with each other. For the relationship between the gene and morphological parameters, NRP1 was correlated with TH and MAP2, while HHEX was correlated with MAP2.

However, a limitation of correlation networks is that they can be confounded by indirect relationships. In contrast, methods that infer the data such as a whole, such as the Bayesian network, includes only direct effects and is considered more biologically interpretable due to removal of indirect correlations [31]. The integrative Bayesian network showed that the node of a transcriptional regulator ID2 was located at the top of the network hierarchy and was positively related to MAP2 (Figure 4B and Figure S4). The enzyme TET2 was not connected to the network, suggesting that the inferred network using a Bayesian algorithm was able to remove indirect relationships. Finally, to verify the biological relevance of the inferred connections, a physiological network was generated based on findings of from previous studies using the Ingenuity Knowledge Base. In accordance with Bayesian network, the physiological network showed that there were no direct relationships between TET2 and the functions of either “Neurogenesis” or “Differentiation of embryonic stem cells”, whereas ID2 was connected to “Neurogenesis” (Figure 4C). It was reported that induction of the ID2 gene expression, which was also observed in hESC-derived sphere after TMD treatment in the present study, increased differentiation of Tuj1 and GFAP-positive neurosphere cells [32]. Three environmental chemicals other than TMD had very limited or no significant changes in neuronal differentiation in this model. BDE47 has recently been reported to have inhibitory effects on human ES-derived neuronal cells similar to our model [21,33]. Similarly, BPA has been found to have suppressive effects on neuronal stem cells [34,35]. Regarding OH-PCB187 or mother compounds of PCB, epidemiological studies suggest a negative relationship with IQ and a relationship with ADHD [36], but no report has been made in vitro. This is the first report of exposure of OH-PCB187 to human ES.

## 3. Materials and Methods

### 3.1. Ethics Statement

Experiments using hESCs were approved by the ethics committees of the National Institute for Environmental Studies in accordance with to the guideline of the Japanese Ministry of Education, Culture, Sports, Science, and Technology (Notification number 20 of Ministry of Education, Culture, Sports, Science and Technology No. 857, 10 October 2008). The experimental plan was registered on the NIES ethics committee (Notification number: 2008-1, Reception number: 2007-1, 15 October 2008).

### 3.2. Chemical Exposures, Culture and Neuronal Differentiation of Human ESCs

Dimethyl sulfoxide (DMSO) and BPA were obtained from Sigma–Aldrich Co. (St. Louis, MO, USA). TMD were obtained from Wako Pure Chemicals (Tokyo, Japan); 4OH-PCB187 and BDE-47 were obtained from AccuStandard (New Haven, CT, USA). DMSO was used as the primary solvent for all chemicals. The final concentrations of DMSO in the media did not exceed 0.1% (*v*/*v*). Human embryonic stem cells (khES3) were maintained and differentiated as described previously [10,11]. The hESC line, KhES-3 (XY genotype), was provided by Dr. Hirofumi Suemori, Research Center of Stem Cells, Institute for Frontier Medical Science, Kyoto University according to the NIES institutional guidelines for the use of human ES research [37]. All experiments using hESCs were approved by the ethics committees of the National Institute for Environmental Studies and the University of Tokyo in accordance with the guidelines of the Japanese Ministry of Education, Culture, Sports, Science, and Technology. The procedures for the maintenance of hESCs were performed as described previously [37,38,39]. MEFs were used as feeder cells for the culture and passage of the hESC line KhES3 in the DMEM/F12 media containing 20% KSR, 100 µM NEAA, 2 mM l-glutamine, 100 µM 2-ME, and 5 ng/mL bFGF. After five times of passages with additional MEFs, the MEFs were eliminated by a brief enzymatic treatment. The hESC colonies left on the dishes were harvested. The hESCs (purity > 99%) were seeded at 9.0 × 10^3^ cells/well in the medium containing DMEM/F12, 20% KSR, 100 µM NEAA, 2 mM l-glutamine, 100 µM 2-ME, and 10 µM of ROCK inhibitor Y-27632 (Day 1). The generated EBs were cultured for 7 days in the medium, which was exchanged every two days, followed by growth in the medium without Y-27632 for two days. The growing EBs were cultured for 2 additional days in NIM containing DMEM/F12: Neurobasal^®^ Medium (1:1), N-2 Supplement, B-27^®^ Supplement, GlutaMAX™-I, Penicillin-Streptomycin to promote neuronal differentiation. Then, EBs were re-plated onto O/L-coated 24-well-plates at 20 EBs/well. They were cultured for 7 days in neuronal proliferation medium (NPM) containing DMEM/F12: Neurobasal^®^ Medium (1:1), two-fold concentrations of N-2 Supplement, two-fold concentrations of B-27^®^ Supplement, GlutaMAX™-I, Penicillin-Streptomycin, 20 ng/mL bFGF. The medium was exchanged every 3 days. hESCs were allowed to form embryoid bodies (EBs) in the round bottom 96-well plate (Falcon 351177). The EBs were seeded onto ornithine–laminin (O/L)-coated 24-well plates to promote neuronal differentiation with the sequential exchange of authentic appropriate neuronal differentiation media every other day. The schedules for the formation of sphere and neuronal differentiation of hESCs are summarized in Figure 1A. Briefly, hESCs were allowed to form sphere in the round bottom 96 well plate for 7 days. Cells were exposed to each chemical from Day 3 to 7 during sphere formation. Doses of chemicals used here were within the clinical dose range for TMD, blood, urinary or breast milk concentrations reported in population studies for BPA, 4OH-PCB187 and BDE-47 [40,41,42,43,44,45]. The spheres were then seeded onto poly-ornithine/laminin 111-coated 24-well plates to promote proliferation and neuronal differentiation for 21 days, and the medium was refreshed every 3 days, as reported previously [11].

### 3.3. Immunocytochemistry and Image Analysis

Differentiated cells on day 28 were immunolabeled with human anti-MAP2 (M4403, 1:200, Sigma-Aldrich) or anti-TH (AB152, 1:200, Millipore, Burlington, MA, USA) antibodies, followed by staining with Alexa 546-conjugated secondary antibody (1:1000, Invitrogen, Carlsbad, CA, USA). The nuclei were stained with Hoechst 33,342 solution (Dojindo, Tokyo, Japan). The values for the area of fluorescent signals were analyzed using an IN Cell Analyzer 1000 (GE Healthcare UK Ltd., Buckinghamshire, UK), as previously reported [11,46]. In brief, immunofluorescent images were automatically acquired using the IN Cell Analyzer to quantify the differences in the cellular nuclei and cellular phenotypes. Fluorescent microphotographs (12 fields per well of a 24-well plate) were obtained automatically. Hoechst-positive nuclei and MAP2-positive or TH-positive neurites were recognized using IN Cell Developer Software. Removal of apoptotic or necrotic cells was performed by measuring the Hoechst-positive nuclei’s morphological features with nuclei fragmentation and chromatin condensation. Viable cells and apoptotic cells were classified according to nuclei size and the nuclei fluorescence signal density. The software was also used to identify neurons that stained positive with both nuclear stain and MAP2 antibodies, and to characterize the neurites extending from these cells. The length of the neurite for each identified cell was measured, respectively. Data were expressed as the mean neurite area per cell. Cells were cultured and stained in 6 independent wells for each condition, 12 fields per well were observed and values were calculated. Statistical analyses for cellular morphology were performed with Excel statistics (Microsoft 2016). All data were expressed relative to the means of the control groups. All results were represented as mean ± standard error (SE). All data were analyzed by one-way analysis of variance (ANOVA), followed by Fisher’s least significant difference (LSD) post hoc test, to compare the effect of each dose with the DMSO control groups. *p*-values less than 0.05 were considered statistically significant.

### 3.4. Microarray Gene Expression Profiling

Four spheres from different experimental groups were pooled together separately. RNAs were isolated from each group on day 7. To detect changes in gene expression in spheres after the chemical exposure, microarray analyses were performed on the RNA sample using a microarray. Total RNA from spheres was isolated with the RNeasy Mini Kit (Qiagen, Hilden, Germany). Fifty nanograms of total RNA pooled from three independent samples was fluorescently labeled and hybridized to Agilent 8 × 60 K-Human Genome Microarrays (Sureprint G3 Human GE 8 × 60 K Ver.2.0 1color 4; Agilent Technologies Inc., Santa Clara, CA, USA). The arrays were hybridized and scanned in accordance with the manufacturer’s directions at the facility of Hokkaido System Science Co., Ltd. (Sapporo, Japan), as reported previously [10]. The raw data was filtered based on signal intensity values and in the lowest 20 percentile and then filtered by FLAG-tag to remove entities that were not detected using GeneSpring GX12.10 software (Agilent Technologies Inc., Santa Clara, CA, USA). The microarray data were submitted to Gene Expression Omnibus (GEO) and registered as GSE151239 [47].

### 3.5. Knowledge-Based Pathway Analysis and Network Analysis

To explore the biological interpretation of the transcriptome data, the canonical pathway analysis, disease and bio-function annotation, and upstream causal network analysis were performed using the knowledge-based functional analysis software, Ingenuity Pathways Analysis (IPA, Ingenuity Systems, Redwood City, CA, USA). In IPA analysis, the fold change is a ratio (case/control), and it is up-regulated as it is between 1 and +infinity, and the value (x) between 0 and 1 is converted as “−1/x”. It is down-regulated with a distribution from −infinity to −1. Correlations of gene expression and morphological measures were calculated according to the Pearson correlation coefficients method in R Bioconductor (https://www.bioconductor.org/). Bayesian network analysis was applied based on using the TAO-Gen algorithm using the web-based RX-Taogen software (http://extaogen.nies.go.jp/). The replicate exchange time was set as 20,000, as reported previously [39]. The networks were visualized using the Gephi software (https://gephi.org/).

## 4. Conclusions

The recent technology using three-dimensional neuronal sphere models derived from pluripotent stem cells has provided new insights into the etiology of neurological diseases and new therapeutic strategies for assessing chemical safety. In this study, we explored the comparative effects of TMD and environmental chemicals such as BDE47, BPA, and 4OH-PCB187 at realistic blood concentration levels on the neuronal differentiation of hESC-derived spheres. In conclusion, exposure to TMD, but not other chemicals, at the early stage of development influenced neuronal differentiation of MAP2-positive and TH-positive neuronal cells from hESC-derived spheres. Transcriptomic analysis and functional annotation at the early stage of neural differentiation showed specific TMD-induction genes associated with the ERK and synaptic signaling pathways. Computational network analysis of genetic and morphological actions of TMD during neuronal differentiation predicted that TMD-induced expression of DNA-binding protein inhibitor ID2 played an important role in neuronal development from hESC-derived spheres. These findings provide direct evidence that early transcriptomic changes during differentiation of hESCs by chemical exposure influenced neuronal development in later stages.

## Supplementary Materials

Supplementary Materials can be found at https://www.mdpi.com/1422-0067/21/15/5564/s1. Figure S1: Effects of chemical exposure on cellular morphologies and *MAP2* gene expression during the neuronal differentiation from human embryonic stem cell (hESC)-derived spheres. Figure S2: Synaptogenesis Signaling Pathway. Figure S3: Upstream causal network. Figure S4: Representative network with probability values in Bayesian network analysis. Networks were generated based on the TAO-Gen algorithm using the web-based RX-Taogen software. Table S1: Lists of the TMD-specific genes selected after the comparison between TMD and each of the three chemicals correspondingly for IPA-pathway analysis. Table S2: Prediction of expression patterns of genes involved in the function of “Differentiation of embryonic cells” by IPA.

## Abbreviations

BDE-47	2,2′,4,4′-tetrabromodiphenyl ether
BPA	bisphenol A
DMSO	dimethyl sulfoxide
hESC	human embryonic stem cell
4OH-PCB187	4-hydroxy-2,2′,3,4′,5,5′,6-heptachlorobiphenyl
TMD	thalidomide
