# Supplementary material for: Early Transcriptomic Changes upon Thalidomide Exposure Influence the Later Neuronal Development in Human Embryonic Stem Cell-Derived Spheres
Source: Int J Mol Sci. 2020 Aug 3;21(15):5564. doi: 10.3390/ijms21155564 (PMC7432054; doi:10.3390/ijms21155564)
Supplement: Supplementary file 1 [file ijms-21-05564-s001.zip › ijms-855204 (Table S1)-resubmit.pdf]

Table S1. Lists of the common TMD-specific genes seselected after the comparison between TMD and each of the three chemicals correspondingly for IPA-pathway analysis

Gene Symbol							
ABCA5	CDH6	FAHD2A	IFI6	LUZP2	ONECUT1	SCIMP	THSD7A
ABLIM2	CES3	FAM124B	IKZF1	MAGI3	OOEP	SCN1B	TIAM2
ACTG2	CFAP70	FAM167A	INSM1	MAJIN	OPRL1	SCNN1B	TM4SF18
AFAP1L2	CFC1/CFC1B	FAM228A	IRX1	MAL	OR4X2	SEC1P	TMEM130
AFMID	CHD5	FAM83A-AS1	ISLR2	MAP2	OR52A1	SEMA3G	TMEM132C
AK4	CKMT2	FAT3	JAKMIP1	MAP6	OSCP1	SGPP1	TNIK
AKR1C1/AKR1C2	CLC	FAT4	JPX	MC1R	P4HA2	SH2D6	TRIM15
ANGPT1	CLDND2	FBN2	KALRN	MCOLN3	PBOV1	SHISA3	TRIM22
ANKRD19P	CLEC4GP1	FBRS	KANSL1L	ME1	PCBP1-AS1	SHOX	TRPC4
ANKRD6	CLIC3	FBXO15	KAZALD1	MED31	PCBP3	SLC16A12	TTC41P
ANXA8/ANXA8L1	CLSTN2	FBXO31	KCNA7	MEIOB	PCDH11X	SLC16A3	TTN
APBA2	CNGA1	FBXO48	KCNH5	MFAP3L	PCDH11Y	SLC22A11	TYRO3P
AQP4	COL12A1	FEZF1	KCNJ2	MISP	PCDH9	SLC24A1	UBE2D3
ARID5B	COL24A1	FGFR3	KCNJ4	MME	PCDHAC2	SLC26A5	USP3
ATP10A	COL5A2	FIGNL2	KCNMA1	MOB3C	PCDHGB2	SLC37A2	VAV1
ATP2A3	CPLANE2	FLRT3	KDM2B	MROCKI	PDE4B	SLC41A2	VSNL1
ATP6V0D1	CPLX3	FMOD	KHDRBS2	MROH1	PHC2	SLC4A4	VSTM2B
BCAR1	CPO	FNDC1	KIAA0408	MSN	PIGZ	SLC9A5	VSX2
BHLHE40	CREB5	FOXA2	KLHDC7A	MT1B	PLEK2	SLC9B1	WIF1
BIRC5	CRHR1	FOXJ1	LBX2	MT1E	PLEKHM3	SLED1	YPEL2
BNIPL	CRTAC1	FRMPD4	LCP2	MT1F	PPIAP46	SMDT1	ZAN
BRD7P3	CRYBA4	FRZB	LDHD	MT1G	PRDM14	SMIM22	ZNF44
BST2	CRYBB1	G0S2	LHX1	MT1H	PRDM6	SNED1	ZNF699
BTN3A1	CTAG1A/CTAG1B	GAD2	LILRB4	MT1HL1	PRDM8	SOBP	ZNF818P
C10orf25	CXXC4	GAGE7	LILRB5	MT1X	PRKCD	SOCS2-AS1	ZNF880
C1QL1	CYP1B1-AS1	GALNT18	LIMCH1	MTRR	PRPH2	SORCS1	ZNRD1ASP
C1QL2	CYP26A1	GALNT9	LINC00112	MTSS1	PSD4	SPAG4	ZXDA
C1QL3	DACT1	GCSAM	LINC00312	MYORG	PTGER2	SPARCL1	
C2CD4D-AS1	DDIT4L	GDPD5	LINC00458	NABP1	PTPRT	SPATA6L	
C3orf35	DDX43	GHRL	LINC01091	NAT8B	PVT1	SRPK3	
C4orf51	DDX58	GJB7	LINC01108	NATD1	PWWP2A	STAB1	
C6orf141	DEF6	GPD1	LINC01501	NCKAP5L	PWWP3B	STMN4	
C9orf64	DENND4C	GPR39	LINC01530	NCR3LG1	RAB26	SUPT3H	
CA4	DGCR10	GRAMD1B	LINC01829	NECTIN4	RABL2A	SUSD4	
CA7	DGKK	GRIA3	LINC02783	NEUROD2	RAMP1	SVIL	
CABP7	DIPK1A	GRIA4	LOC100288966/POTED	NFKBIZ	RAP1GAP2	SYT4	
CACNB4	DMRT1	GRIN2A	LOC100506282	NFXL1	RASSF10	SYTL1	
CALN1	DPPA5	GUCA1B	LOC101926907	NIPAL1	RASSF3	SZT2	
CAPN6	EBF3	GUCD1	LOC101927857	NLRP12	RGS7	TAS2R5	
CARD6	EDNRB	H1-6	LOC101929333	NNAT	RHOU	TBC1D22A	
CCBE1	EEF1AKMT4-ECE2	H2AC2P	LOC105373042	NODAL	RIC1	TCAIM	
CCDC141	EGR4	HCN1	LOC107983985	NOX4	RIPPLY3	TCAM1P	
CCDC149	ELMOD1	HES2	LOC107985840	NPFFR2	RNA5-8SN5	TCTE3	
CCDC80	EPHB1	HFM1	LOC107986268	NPPB	RNF180	TEK	
CCK	ERCC6L2	HHEX	LOC642648	NPY1R	RSPH10B/RSPH10B2	TENM4	
CCN3	ERVMER34-1	HHLA1	LOC729291	NRP1	RUNX1	TENT5A	
CCNE2	ESYT2	HPGD	LOXL2	NUGGC	S100A11	TET2	
CCNG2	EVX2	HTR6	LRRC42	NUP58	S100A16	TFAP2E	
CCR7	F10	ID2	LRRC4C	OC90	S1PR5	TFF3	
CD300A	F12	IDI2-AS1	LRRC72	OLIG3	SCD5	TFPI	
