# Supplementary material for: Early Transcriptomic Changes upon Thalidomide Exposure Influence the Later Neuronal Development in Human Embryonic Stem Cell-Derived Spheres
Source: Int J Mol Sci. 2020 Aug 3;21(15):5564. doi: 10.3390/ijms21155564 (PMC7432054; doi:10.3390/ijms21155564)
Supplement: Supplementary file 1 [file ijms-21-05564-s001.zip › ijms-855204 (Table S2)-resubmit.pdf]

Table S2. Prediction of expression patterns of genes involved in the function of “Differenti

Gene Symbol	Type	Expression Fold Change (TMD/DMSO)	Prediction based on measurement direction
TET2	enzyme	3.08	Increased
HHEX	transcription regulator	2.00	Increased
ID2	transcription regulator	2.30	Increased
NRP1	transmembrane receptor	2.28	Increased
NPY1R	G-protein coupled receptor	0.45	Affected
NODAL	growth factor	0.39	Affected
COL12A1	other	2.82	Affected
FRZB	other	3.84	Affected
KDM2B	enzyme	0.46	Increased
FOXA2	transcription regulator	0.39	Decreased
RUNX1	transcription regulator	0.48	Decreased
IL6ST	transmembrane receptor	0.49	Increased
ANGPT1	growth factor	0.28	Decreased

ation of embryonic cells” by IPA.

Privouse Findings in Ingenuity Knowledge Base
Increases
Increases
Increases
Increases
Affects
Affects
Affects
Affects
Decreases
Increases
Increases
Decreases
Increases
