# Supplementary figures and images for: Early Transcriptomic Changes upon Thalidomide Exposure Influence the Later Neuronal Development in Human Embryonic Stem Cell-Derived Spheres
Source: Int J Mol Sci. 2020 Aug 3;21(15):5564. doi: 10.3390/ijms21155564 (PMC7432054; doi:10.3390/ijms21155564)

**FigS1**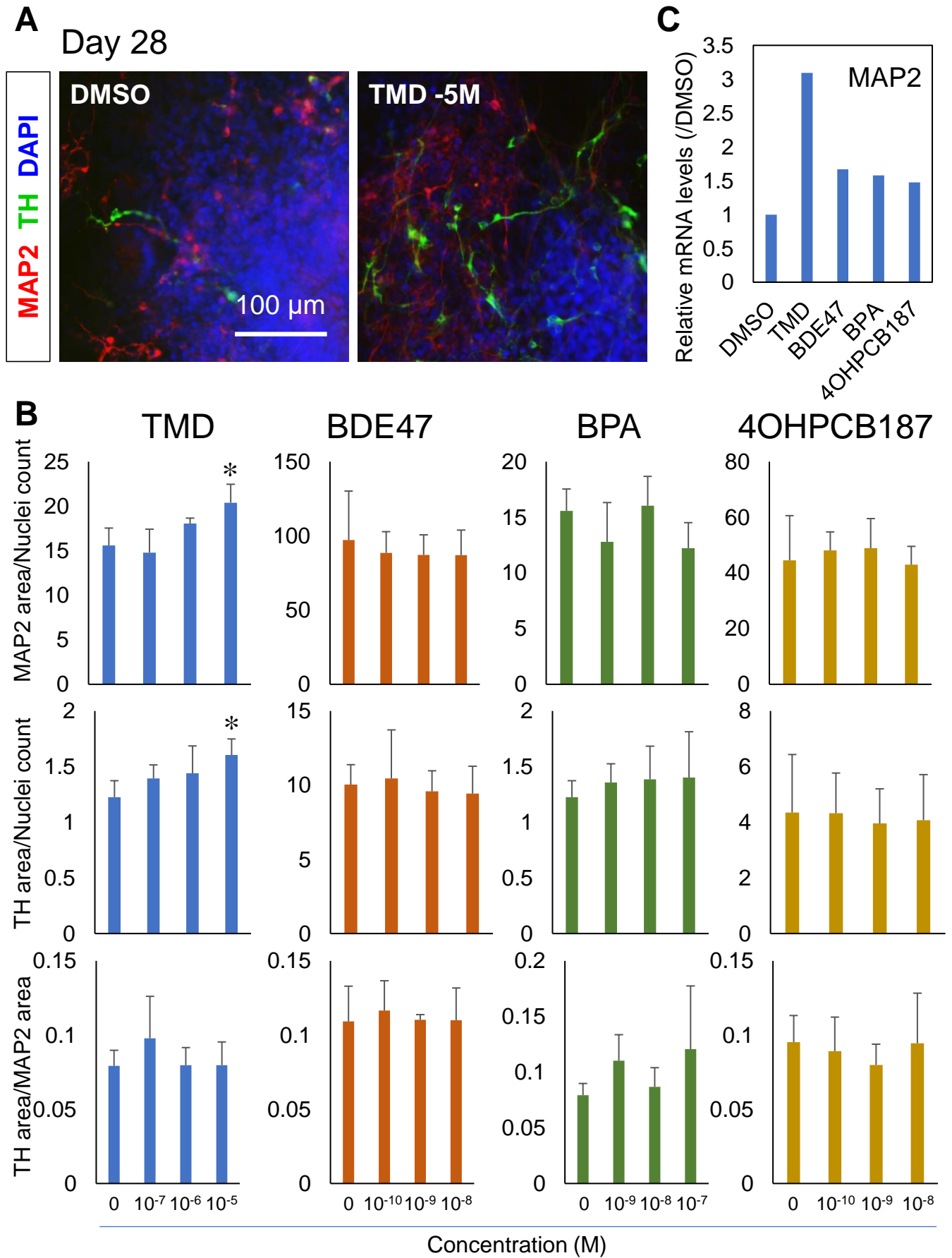

**FigS2**

# Synaptogenesis Signaling Pathway

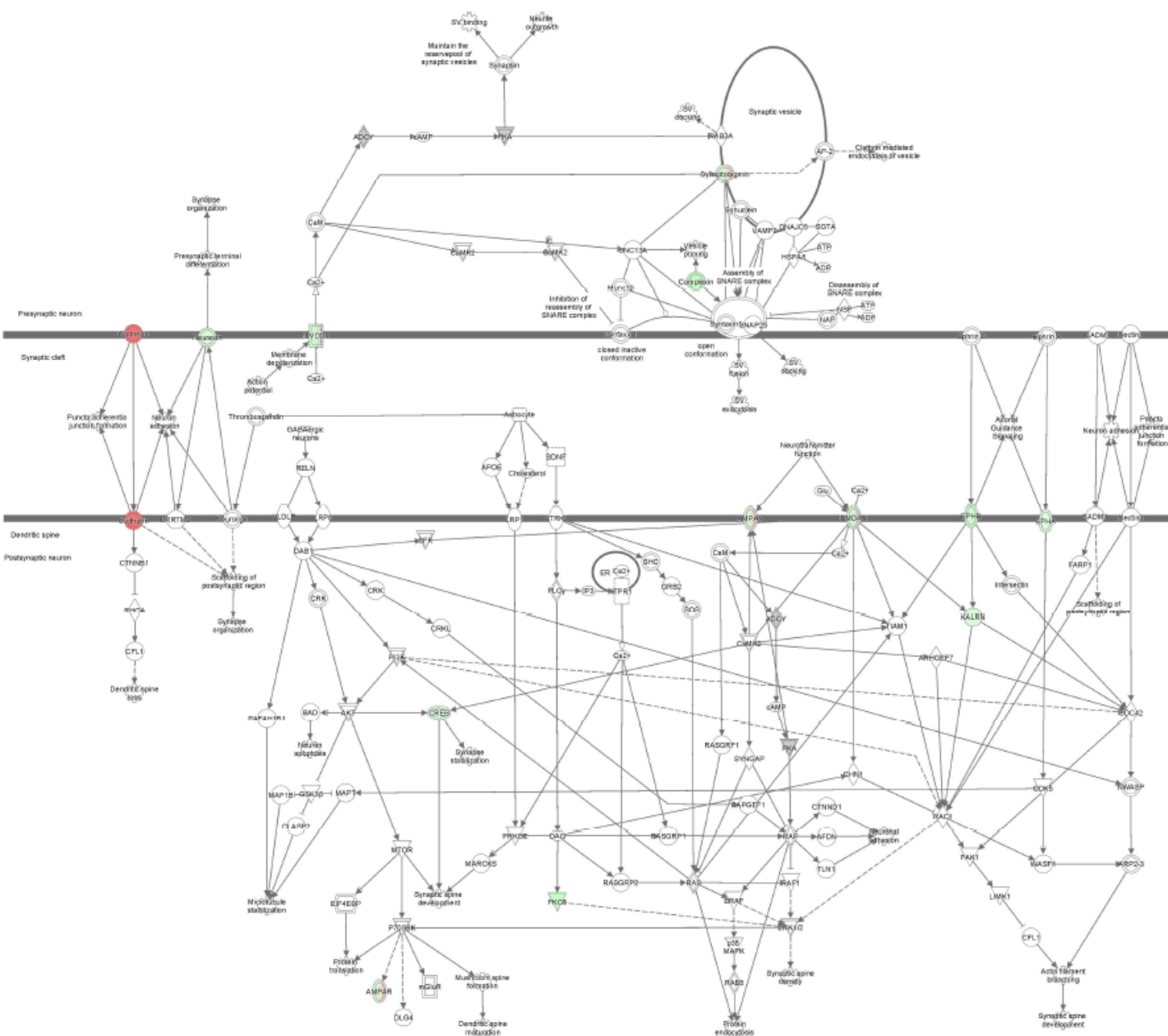

FigS3

Upstream causal network

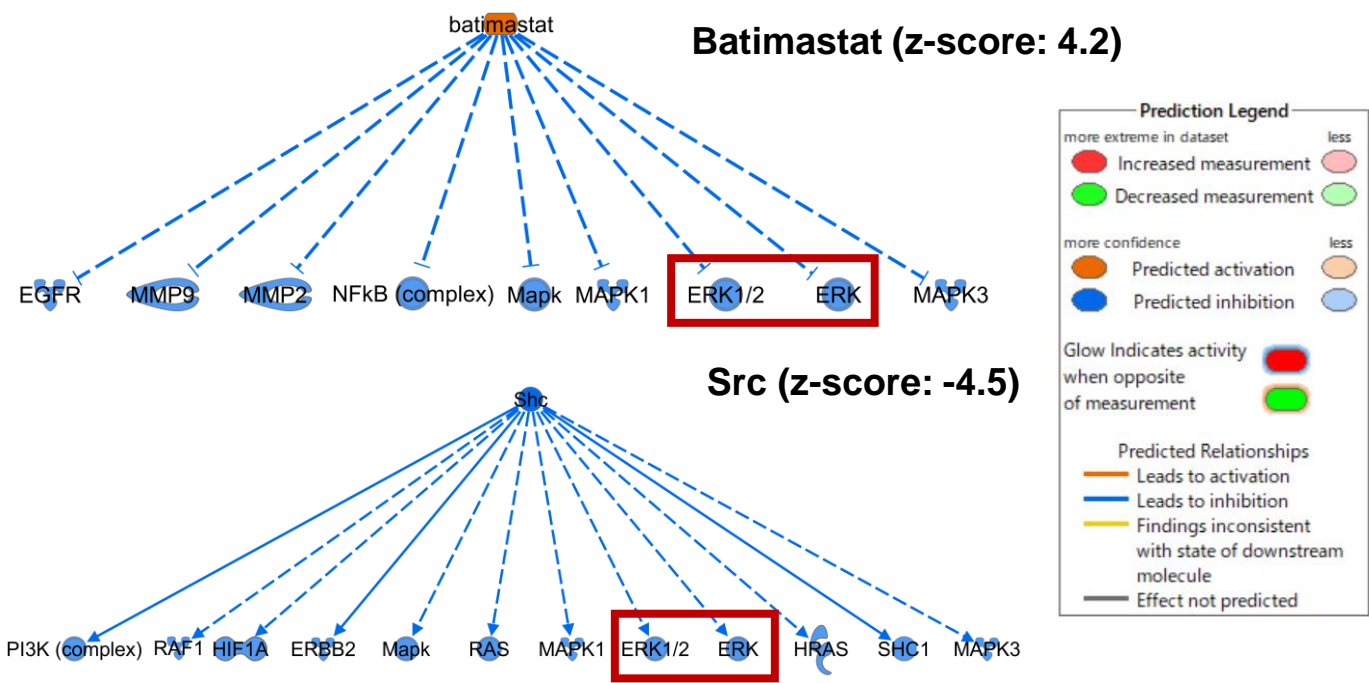

FigS4

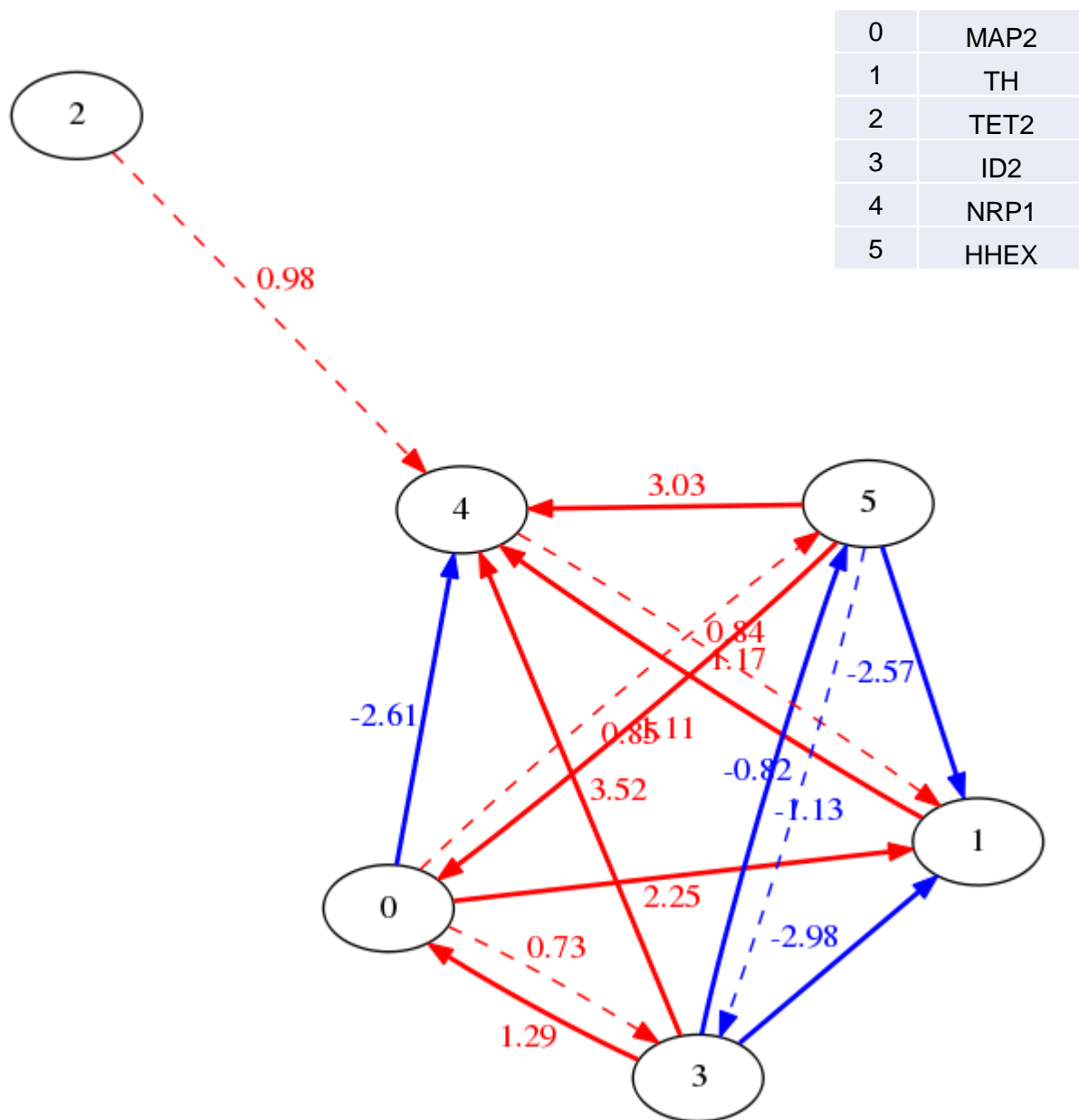

Supplement: Supplementary file 1 [file ijms-21-05564-s001.zip › ijms-855204 (Figures S1-3)-resubmit.pdf]
